# The Chronobiology of Hormone Administration: “Doctor, What Time Should I Take My Medication?”

**DOI:** 10.1210/endrev/bnaf013

**Published:** 2025-04-19

**Authors:** Elena Colonnello, Andrea Graziani, Rebecca Rossetti, Giacomo Voltan, Davide Masi, Carla Lubrano, Stefania Mariani, Mikiko Watanabe, Andrea Marcello Isidori, Alberto Ferlin, Lucio Gnessi

**Affiliations:** Department of Experimental Medicine, Section of Medical Pathophysiology, Food Science and Endocrinology, Sapienza University of Rome, 00161 Rome, Italy; Chair of Endocrinology and Medical Sexology (ENDOSEX), University of Tor Vergata, 00133 Rome, Italy; Unit of Andrology and Reproductive Medicine, University Hospital of Padova, 35128 Padova, Italy; Department of Experimental Medicine, Section of Medical Pathophysiology, Food Science and Endocrinology, Sapienza University of Rome, 00161 Rome, Italy; Endocrinology Unit, Department of Medicine, University of Padova, 35122 Padova, Italy; Department of Experimental Medicine, Section of Medical Pathophysiology, Food Science and Endocrinology, Sapienza University of Rome, 00161 Rome, Italy; Department of Experimental Medicine, Section of Medical Pathophysiology, Food Science and Endocrinology, Sapienza University of Rome, 00161 Rome, Italy; Department of Experimental Medicine, Section of Medical Pathophysiology, Food Science and Endocrinology, Sapienza University of Rome, 00161 Rome, Italy; Department of Experimental Medicine, Section of Medical Pathophysiology, Food Science and Endocrinology, Sapienza University of Rome, 00161 Rome, Italy; Department of Experimental Medicine, Section of Medical Pathophysiology, Food Science and Endocrinology, Sapienza University of Rome, 00161 Rome, Italy; Unit of Andrology and Reproductive Medicine, University Hospital of Padova, 35128 Padova, Italy; Department of Experimental Medicine, Section of Medical Pathophysiology, Food Science and Endocrinology, Sapienza University of Rome, 00161 Rome, Italy

**Keywords:** circadian rhythm, chronotypes, chronobiology, pharmacotherapy, hormone replacement, glucocorticoids

## Abstract

Pharmacotherapy involving hormones and hormone-derived molecules has various potential treatment targets. This includes addressing (partial) hormonal deficiencies, pursuing osteoanabolic effects, providing contraceptive options, or supporting gender-affirming transitions. In chronotherapy, the timing of the administration of active ingredients and different pharmaceutical forms is leveraged to maximize therapeutic efficacy while minimizing adverse effects, based on the principle that it is optimal for drugs to be administered according to the body's circadian rhythms. Just as a drummer sets the pace and keeps the rhythm steady for the entire band, the physician, through the application of chronotherapy, ensures the treatment regimen is harmonized with the body's internal clock. However, while this is a consolidated aspect for several endocrine treatments, for others, it represents a novelty. The new advancements in the treatment of osteoporosis, with the latest parathyroid hormone–related protein analogue, abaloparatide, or in congenital adrenal hyperplasia with the new long-lasting hydrocortisone formulation, are notable examples. We herein summarized the state of the art regarding the hormonal circadian rhythm to discuss in depth the evidence available regarding the correct timing of commonly administered hormonal therapies in adult patients. By offering clear indications, this manuscript delves into the importance of harmonizing hormonal therapy with circadian rhythms through chronotherapy, exploring its potential to enhance therapeutic outcomes while minimizing adverse effects.

## Graphical Abstract

**Graphical Abstract:**
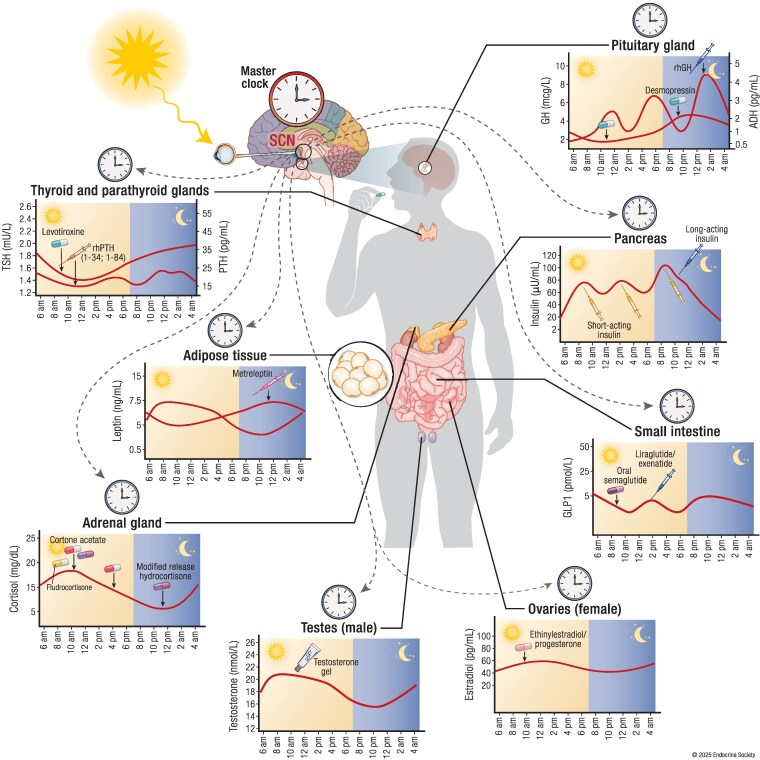

Essential Points

- Chronotherapy in endocrinology leverages circadian rhythms to optimize hormonal therapy, enhancing efficacy and reducing side effects.
- Emerging research highlights the critical interplay between circadian rhythms and metabolic processes, with implications for managing conditions like diabetes and obesity.
- Different hormones, such as cortisol, antidiuretic hormone, and testosterone, demonstrate distinct diurnal patterns that can inform optimal administration timing.
- Recent advances in long-acting hormone formulations have partially shifted the focus from chronotherapy, balancing patient compliance with therapeutic precision.
- Further studies are needed to clarify optimal administration times and enhance the clinical application of chronotherapy.

The endocrine and nervous systems are strongly interconnected, with the hypothalamus serving as a key anatomical and functional link. The hypothalamus regulates the activity of the pituitary gland by producing hormone-releasing factors, whose timing is controlled by the bilateral suprachiasmatic nuclei (SCN). The SCN, regarded as the body's “master clock,” regulate numerous biological functions, including endocrine activity, on a nearly 24-hour cycle that is synchronized primarily by photic transmission to the retina and thus by the alternation of the light/dark cycle (1, 2). At the molecular level, the mammalian circadian master clock in the SCN is structured around a transcriptional-translational feedback loop. Central to this system are the CLOCK and BMAL1 transcription factors, which regulate rhythmic gene expression. They activate the *Per* and *Cry* genes, whose products later inhibit their own gene expression, creating a self-regulating cycle (3). This rhythm is not just centralized in the SCN but also disseminated through “peripheral clocks,” which are present in most tissues and cells within the body (4). These peripheral clocks drive processes like hormone secretion and behavioral functions, including, among others, sleep-wake and feeding-fasting cycles, as well as physical activity. Evidence from both in vitro and in vivo studies supports this complex interplay between master and peripheral clocks (5, 6).

This circadian system, named from the Latin phrase “*circa diem*,” meaning “*about a day*,” is thought to represent an evolutionary adaptation in many organisms to the daily rotation of Earth around its axis (7). Possessing an internal coordination of biological activity the circadian rhythm allows organisms to respond in a proactive, rather than reflexive, manner, to the predicable cycle of alternating light and dark. This seems to confer an advantage in terms of Darwinian fitness, as natural selection operates against organisms with an internal rhythm that is not over 24 hours (8). While the term *diurnal* is used to refer to rhythms that cycle once daily, to be classified as *circadian*, there are precise criteria to be met. First, the rhythm must have a period of nearly 24 hours, which can be observed even in the absence of time cues, thanks to the activity of the master clock, which keeps it constant (9). Second, the rhythm must remain nearly constant in the presence of changes in ambient temperature, a phenomenon known as temperature compensation (10). Third, because the internal rhythm of the master clock is close to, but not precisely, 24 hours, it undergoes a resetting process known as *entrainment* (11) in response to external time cues such as light, temperature, and food, which are collectively referred to as “entrainment factors” or “zeitgeber” (12).

Chronobiology delves into how living organisms adapt their biological activities to solar rhythms, influencing the timing and duration of these activities (13, 14). As accumulating evidence suggests that a plethora of human health problems are related to dysfunction or desynchrony of the circadian system (15), this field of research appears particularly intriguing and challenging.

Chronotherapy represents a therapeutic strategy designed to synchronize the administration of pharmacological agents with the body's inherent circadian rhythms, in order to maximize therapeutic efficacy while concurrently reducing the potential for adverse effects. This approach employs a variety of strategies, including designing pharmaceutical formulations whose pharmacodynamic and pharmacokinetic profiles are strategically timed to coincide with the body's inherent biological rhythms (16, 17). Indeed, strands of research have already demonstrated the circadian variability in the pharmacokinetics (PK) and pharmacodynamics (PD) of drugs, indicating that the time of administration influences both therapeutic outcomes and adverse effects (18-20). Rhythmic variations in the physiology and gene expression of different tissues can affect drug actions, including pharmacodynamic responses, based on the availability or function of target proteins (21). The timing of drug administration could therefore play an important role in determining key pharmacokinetic parameters, including overall exposure (AUC), bioavailability (F), clearance (CL), peak concentration (C_max_), peak exposure time (t_max_), as well as half-life (t_1/2_) (9, 22, 23). An emblematic example is that of short-acting statins, which are to be given at bedtime considering that the rate-limiting enzyme of cholesterol biosynthesis, 3-hydroxy-3-methylglutaryl coenzyme A reductase, peaks at night in humans, hence a more efficient inhibition of cholesterol biosynthesis process is possible at this time (24). Another relevant chronotherapeutic strategy is to integrate into the clinical management the patients’ chronotypes—an individual's natural preference for being active and alert during specific times of the day that corresponds essentially to the body's internal clock or circadian rhythm (25). Chronotypes are intrinsically determined but also influenced by environmental factors, such as work schedules and lifestyle habits. When the chronotype is misaligned with the daily schedule, it can lead to sleep disturbances, reduced productivity, as well as possibly reduced compliance with medication (26, 27). In the last 10 years, some studies have highlighted the importance of tailoring medical treatments to an individual's chronotype. For instance, the Chronotype sub-study cohort of the Treatment in Morning vs Evening (TIME) study examined the effect of timed dosing of antihypertensive medications according to patient chronotype on cardiovascular outcomes, suggesting that alignment of dosing time of usual antihypertensives with personal chronotype could lower the incidence of nonfatal myocardial infarction compared to a “misaligned” dosing time regimen (28). Additionally, research indicated that evening chronotypes may experience reduced efficacy when treated with selective serotonin reuptake inhibitors (SSRIs) (29). Furthermore, the PK of metformin has been shown to vary significantly depending on the time of day it is administered, forecasting a potential role for chronomodulation in the therapy of type 2 diabetes (T2DM) (30). These findings suggest that considering a patient's chronotype can enhance the effectiveness of medical treatments, which fosters the importance of personalizing the treatment to achieve better outcomes.

From an endocrine perspective, numerous hormones display distinct circadian rhythms, with their secretion and activity tightly regulated by the body's internal biological clock (Fig. 1) (31). We herein examine the circadian rhythms of key hormones used and explore the application of chronotherapy in hormone therapies, assessing the optimal timing for administration based on diurnal secretion patterns and available evidence in the adult population.

**Figure 1.**
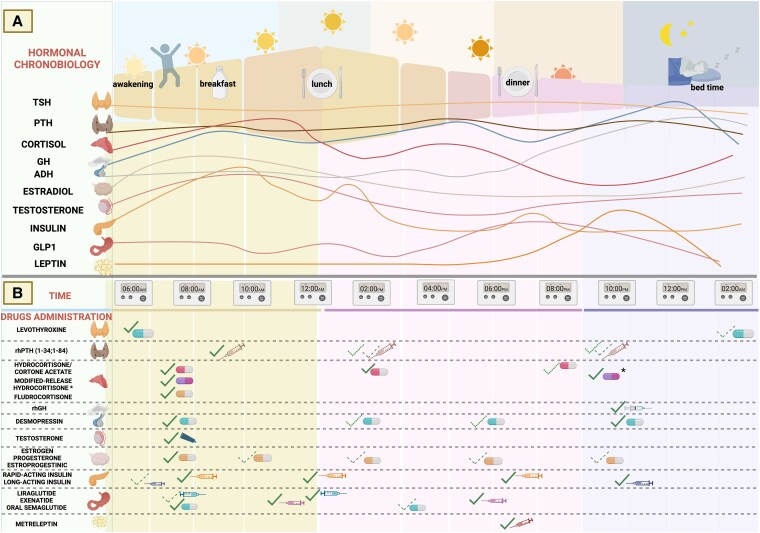
(A) Diurnal rhythm of thyroid-stimulating hormone (TSH), parathyroid hormone (PTH), cortisol, growth hormone (GH), antidiuretic hormone (ADH), estradiol, testosterone, insulin, glucagon-like peptide 1, and leptin. (B) Timing of hormone administration, including levothyroxine, recombinant human parathyroid hormone (rhPTH), hydrocortisone, cortisone acetate, modified-release hydrocortisone, fludrocortisone, recombinant human growth hormone (rhGH), desmopressin, testosterone, estrogen, progesterone, estroprogestinics, rapid-acting insulin, long-acting insulin, liraglutide, exenatide, oral semaglutide, and metreleptin. Legend: Solid ✓ indicates recommended administration, dashed ✓ indicates alternative administration, and dotted ✓ indicates additional administration. Timing of hormone administration is indicative; treatment should be personalized based on the patient's wake/sleep schedule and chronotype for optimal efficacy. *Bedtime administration of the modified-release hydrocortisone applies only to congenital adrenal hyperplasia (CAH). Created in BioRender (https://BioRender.com/j78z959).

## Thyroid Hormones

### Thyroid Hormones Circadian Rhythm

The hypothalamic–pituitary–thyroid axis activity follows a circadian rhythm, mainly under the control of the central circadian pacemaker in the SCN (32). Moreover, the existence of an independent activity of the intrinsic thyroid clock has been supported by animal studies (33). Plasma concentration of thyroid-stimulating hormone (TSH) begins to rise in the late afternoon or early evening, with a nocturnal surge before sleep onset, and then it declines (Fig. 1A). On the contrary, from the limited data available, thyroxine (T4) and triiodothyronine (T3) have been reported to show a morning peak and an evening or nighttime nadir, with free-T3 (fT3) levels peaking approximately 90 minutes after TSH levels (34). This diurnal variation is affected by glucocorticoid secretion/administration, melatonin, and thyroid hormones themselves (35, 36). On top of that, a low-amplitude ultradian rhythm (a rhythm at high frequencies with a period <24 hours) exists and is affected by sleep patterns, seasonal changes, and food intake. Indeed, studies have shown that acute nocturnal sleep deprivation causes TSH secretion to rise (37), causing higher morning plasma TSH levels, while partial sleep loss leads to modest but statistically significant declines in TSH and free T4 (fT4), mainly in female individuals (38). Furthermore, TSH secretion appears to decrease during summer and increase during winter, correlating with daily temperatures (39), as cold activates the catecholaminergic system, thereby causing thyrotropin-releasing hormone (TRH) release (which also explains the sudden increase in TSH in newborns that returns to its initial value within 48 hours) (40). Chronic shift work, jet lag, and irregular sleep-wake cycles may disrupt the circadian rhythm, increasing the risk of thyroid disease (41). TSH rhythmicity is also often disrupted in patients with thyroid disorder (32, 42, 43): in cases of hypothyroidism, particularly when severe, the secretory regularity of TSH is markedly decreased (44).

However, while in clinically overt hypothyroidism, particularly elevated TSH concentrations often suppress the observable daily secretion pattern of the thyroid hormones, individuals with subclinical hypothyroidism, with only mildly elevated TSH levels, may retain a daily secretion rhythm similar to normal (44). For central hypothyroidism, particularly the cases resulting from thyrotropin-releasing hormone (TRH) deficiency or cranial irradiation for brain tumors, the TSH rhythm is often disrupted or absent (32). Similarly, in patients with hyperthyroidism, the TSH rhythm is obliterated, primarily due to alterations in the feedback regulation system (32). However, while subjects with suppressed TSH due to Graves’ disease or to activating TSH mutations have a blunted TSH rhythm, in patients with thyrotropinomas, TSH diurnal secretion pattern is preserved, although more irregular (45).

### Thyroid Hormones Therapy

The TSH circadian rhythm seems to be maintained in patients treated with T4 and T3; however, there is no clear evidence on the best timing of supplementation. Current preparations of T4 and T3 mainly have an oral bioavailability, although intravenous (46), intramuscular (IM) (47), and even rectal administration (48) are available for patients who are unable to ingest or absorb oral medication. The oral preparations are administered on an empty stomach, 30 to 60 minutes before a meal to prevent food or medications from interfering with their uptake. Differences in pharmacokinetic properties exist among the various formulations, that is, tablet vs liquid or soft gel capsule. However, on average, they result in immediate hormone release, with an increase in fT4 levels within the first 2 hours after administration. According to the American Thyroid Association (ATA), levothyroxine (L-T4) should be taken either 60 minutes before breakfast or at bedtime (3 or more hours after the evening meal), for optimal, consistent absorption (49). Nevertheless, comparative studies on morning vs evening administration of levothyroxine reported conflicting results. One study in Muslim patients during Ramadan, where L-T4 administration before breaking the fast at sunset (*iftar*) was compared to that prior to an early morning meal before sunrise (*suhoor*), did not report any statistically relevant difference (50). On the other hand, a later study claimed that post-iftar or pre-suhoor L-T4 intake significantly altered TSH levels, whereas pre-iftar did not (51).

According to a 2020 meta-analysis of 10 studies, L-T4 administration at bedtime is as effective as administration before breakfast, with no significant difference in the TSH level (SMD = 0.09; 95% CI: −0.12, 0.30; *P* = .39) despite higher FT4 levels if administered at bedtime (SMD = −0.27; 95% CI: −0.52, −0.02; *P* = .03) (52). As adherence itself is the major factor responsible for the therapeutic efficacy of L-T4, bedtime administration may be an appealing option to many, provided that dinner and bedtime are not too close. In addition, there are a number of foods and drugs known to impair L-T4 absorption: this occurs due to either chelation, such as in the case of calcium salts, iron, sucralfate, orlistat, bile acid sequestrants, fiber and soy products, or to inhibition of solid tablets dissolution, through interfering with gastric pH, such as with antacids, proton pumps inhibitors, and coffee (53). Concomitant or near L-T4 administration, in these cases, is to be avoided (54). Given the described morning peak of endogenous T4, and that T4 levels transiently increase for about 9 hours after assuming oral L-T4 (55), it is reasonable to presume that morning administration better mimics physiology. Therefore, if the patient deems equally practical bedtime or morning administration, the latter should be encouraged (Fig. 1B). Given the described rhythmicity, and the interference with food (56), although a consensus regarding the appropriate timing for sample collection for thyroid hormones is currently lacking (57), a morning plasma sampling in a fasting state, before taking the therapy (58), is suggested to assess thyroid hormones levels (59, 60) (Table 1).

**Table 1.** Clinical recommendations for hormone administration timing in relation to blood sampling

Hormone	Timing of blood collection	Fasting	Hormonal therapy administration	Timing of hormonal therapy
***TSH, FT3, FT4***	Morning (recommended (49))	Suggested	After blood collection (recommended (49, 58))	Soon after (recommended (49, 58))
***PTH***	Morning (recommended (61))	Recommended (61)	N/A	N/A
***Cortisol***	Early morning (recommended (58, 62))	Suggested	After blood collection (for adrenal insufficiency—to check hormonal concentration at nadir—recommended (62))	Upon awakening (for adrenal insufficiency—recommended (62))
***Testosterone***	Morning (recommended at 8-10 Am (58, 63, 64))	Recommended (63, 64)	After blood collection (to check hormonal concentration at nadir—suggested) Before blood collection (to check absorption—suggested)	Morning (to check hormonal concentration at nadir- suggested) 2 hours before blood collection (to check absorption—suggested)
***IGF-1***	Morning (suggested)	Suggested	Before blood collection (suggested)	N/A

“Recommended” indicates timing or indications supported by published guidelines or authoritative expert opinions (see references), while “suggested” reflects our proposal based on the available evidence from the literature (see main text for details).

Abbreviations: FT3, free triiodothyronine; FT4, free thyroxine; IGF-1, insulin-like growth factor 1; PTH, parathyroid hormone; TSH, thyrotropin (thyroid-stimulating hormone).

Regarding L-T3, the use of either standard liothyronine or sustained-release triiodothyronine as monotherapy is not recommended (49), nor is its combination with L-T4 (65, 66). However, the combination therapy of the two could be considered in compliant L-T4-treated hypothyroid patients who have persistent complaints despite serum TSH values within the reference range, based on the assumption that euthyroidism can be reached simultaneously in all tissues of hypothyroid patients only by L-T4 + L-T3 in a dose ratio that mimics the physiological T4-to-T3 secretion ratio by the human thyroid gland (which is close to 13:1 by weight) (67). If L-T4 can be given once daily, the daily L-T3 dose should be divided –if possible- into 2 doses, one before breakfast and the largest one before sleeping (68), for a maximum dosage of 60 to 80 µg/day. The rationale for giving L-T3 twice daily (or even more) is the relatively short t_1/2_ of L-T3, the peak serum T3 values occurring at 2 to 4 hours after ingestion, and a physiological diurnal variation in serum T3 with the peak around 4 Am and the nadir between 3 and 5 Pm (69).

## Parathyroid Hormone

### Parathyroid Hormone Circadian Rhythm

Parathyroid hormone (PTH) shows a precise circadian rhythm, with a small peak occurring between 4 and 7 Pm, followed by a broader, longer-lasting increase from late evening to early morning, another peak between 2 and 6 Am and a late morning decrease (70-73) (Fig. 1A), but how the SCN regulates PTH secretion is unknown (70). Fasting decreases levels of PTH and abolishes or reduces the circadian rhythm and nighttime peak (74). Moreover, it has been proven that PTH, like other hormones, has seasonal fluctuations, declining about 20% below the annual mean during the summer and thus reflecting seasonal rhythms of vitamin D and bone turnover markers (73). The nocturnal peak appears to be earlier and greater in men than women (75). Other than this tonic and circadian dynamic, pulsatile PTH secretion occurs, accounting for about 25% of the total amount of PTH secreted, with small amplitude pulses that can occur as often as 10 to 20 minutes with a frequency of 6 to 7 pulses per hour (76). When PTH is secreted in its physiological pulsatile manner, an osteoanabolic effect is seen, in contrast to the effect of continuous PTH secretion. Indeed, it was proven that patients with primary hyperparathyroidism (PHTP) lose the circadian rhythm for PTH (77, 78), and continuous PTH secretion has also resorptive effects, predominantly in cortical bone, although with a partial preservation of trabecular bone mass and microarchitecture (79). This finding not only gave some evidence for a degree of anabolic action of PTH in the trabecular compartment of bone, but it also provided a rationale for the therapeutic development of PTH for bone disorders (76, 77).

### Parathyroid Hormone Therapy

PTH is available pharmaceutically as rhPTH (1-84) and as rhPTH (1-34). While the former is the analogue of the entire PTH molecule, the latter corresponds to its biologically active portion, being the first 34 amino-terminal residues. Both are administered as subcutaneous injections, and the t_1/2_ of each form is 2.5 and 1 hour, respectively (76). Studies on rodents treated with intermittent or continuous PTH infusion confirmed the hypothesis that the osteoanabolic effects of PTH are obtained with a brief and intermittent administration, rather than a continuous exposure (80). This laid the foundation for the design and the approval of teriparatide (rhPTH 1-34), given once daily at the dosage of 20 μg for the treatment of osteoporosis (81). In chronic hypoparathyroidism, conversely, studies that compared twice-daily rhPTH (1-34) injections to once-daily subcutaneous injections demonstrated that the first maintained mean serum calcium in the low or just below the normal range with normal urine calcium excretion and renal function, proving better than single daily injection (82, 83) (Fig. 1B). However, since PTH physiology involves frequent low-amplitude pulses superimposed on an underlying circadian rhythm, it was hypothesized that several daily injections could mimic more closely the endogenous secretion. Further studies showed that, when using an insulin pump to deliver rhPTH (1-34), as the frequency of PTH injections increased, the total daily dose required to maintain normal calcium homeostasis decreased, leading to the normalization of key biochemical markers of calcium homeostasis (84). The development of pump-delivered rhPTH (1-34) would, in fact, represent an important advance in the replacement therapy of chronic hypoparathyroidism, as it represents the only alternative that has been proven to achieve a physiologic biochemical profile. Nowadays, the use of teriparatide for chronic hypoparathyroidism not adequately controlled by the standard therapy has been approved by the European Medicines Agency (EMA) (85) (86), with a 36 months maximum administration having been authorized, in 1 to 4 injections daily injections to better mimic the tonic state of a functional parathyroid gland (82). Indeed, in light of the different PK properties, rhPTH (1-34) requires at least twice daily injections to guarantee a longer binding to the receptor, whereas rhPTH (1-84) can be used successfully with a once-daily dosing regimen for this purpose (76). Further characterization of the molecular mechanisms of the PTH and parathyroid hormone–related peptide (PTHrp) on PTH/PTHrP receptor interactions led to the development of a PTHrP analogue, called abaloparatide, that interacts preferentially with the transiently linked state of the receptor, having an even better osteoanabolic effect at the single daily dose of 80 μg, a T_max_ of 45 minutes, and a t_1/2_ of 2.3 hours (87). Oral and transdermal routes are currently under study, to approximate the normal secretory physiology of PTH with tonic, circadian, and pulsatile secretory dynamics.

Currently, there are only a few indications about the right timing of the day for PTH administration: a 12-month-long clinical investigation in postmenopausal women reported that morning administration of teriparatide resulted in higher spine bone mineral density compared to evening application (88). Considering these data and the previously reported evidence, especially in the case of therapy with once-daily PTH-recombinant therapy, one early morning administration may be recommended in order to respect the putative physiologic circadian rhythm of PTH serum levels (Fig. 1B). Clinical evidence resulting from findings systematically reviewed in 2013 provides a weak recommendation on the timing of PTH blood sampling (10 Am to 4 Pm), derived from one study only that employed first- or second-generation PTH assays (89). An expert opinion from 2016 recommends that a blood sample for PTH measurement should be obtained in the morning (61). Fasting state is not required, but recommended, also given the influence, albeit small, of food intake on PTH (90, 91), as well as calcium and phosphate levels (92) (Table 1).

## Glucocorticoid and Mineralocorticoid Hormones

### Glucocorticoid and Mineralocorticoid Hormones Circadian Rhythm

**Figure 2.**
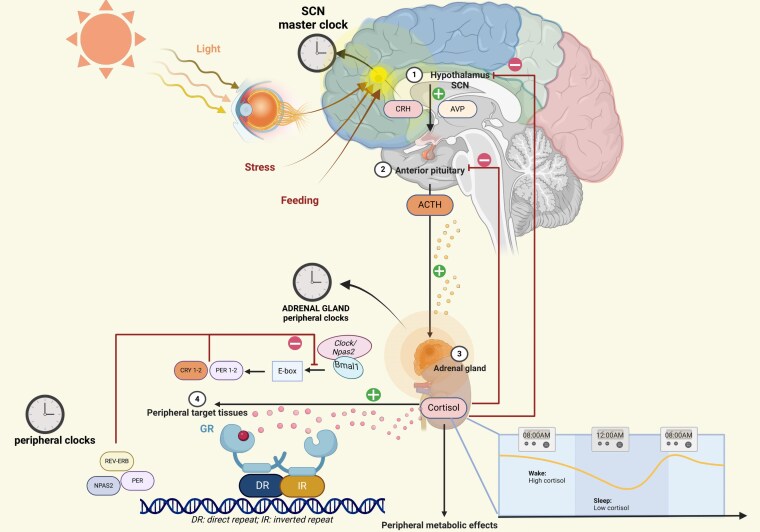
The hypothalamic-pituitary-adrenal (HPA) axis is regulated by the master circadian clock in the suprachiasmatic nucleus (SCN), which synchronizes with light signals via the retino-hypothalamic tract. The SCN influences the paraventricular nucleus (PVN) to release CRH and AVP, stimulating ACTH secretion from the pituitary and driving glucocorticoid production in the adrenal gland. Local adrenal clocks also modulate ACTH responsiveness. Glucocorticoid levels peak before the active phase, and stress signals further activate the HPA axis via inputs to the PVN. Inset: The molecular circadian clock involves a transcriptional-translational feedback loop (TTL) of CLOCK/NPAS2, BMAL1, PERs, and CRYs, cycling over 24 hours. Created in BioRender (https://BioRender.com/s51x907).

The diurnal rhythm of glucocorticoids (GC) is very sensitive to external and endogenous stimuli and is finely controlled by the hypothalamic SCN, showing a circadian regulation. In turn, GC exert strong entraining signals for peripheral circadian oscillators and may feedback on central oscillators, playing a role in synchronizing the cell-autonomous clocks in the human body (93-95). Some evidence of the strict connection between GC and the regulation of the circadian rhythm is provided by the interplay of GC and the clock genes as summarized in Fig. 2. Indeed, GC may influence the transcription of specific clock genes by interacting with glucocorticoid response elements (GREs) within their promoters. Notably, a positive GRE has been identified as a mediator of PER1 induction in response to glucocorticoid (96). Interestingly, BMAL1 knock-out mice exhibited a disruption of the normal glucocorticoid rhythms, suggesting a bidirectional relationship between GC and the clock genes (97). In normal conditions and healthy individuals having stable sleep-wake cycles, levels of serum cortisol start to rise between 3-4 Am, reaching the peak upon awakening between 7-9 Am. During the day, cortisol serum levels progressively decrease, with the nadir being observed at midnight (98, 99) (Fig. 1A). Considering its circadian rhythm, the suggested timing to perform cortisol blood sampling, according to the guidelines, is in the early morning (62). Considering the potential influence on the cortisol secretion reported after meals and several nutrients’ assumption (100, 101) fasting may be preferred (Table 1).

Over the last years, circadian cortisol rhythm disruption has emerged as a risk factor for metabolic and cardiovascular disorders. Indeed, several studies evaluated people without endocrine diseases, analyzing the relationship between disrupted circadian cortisol rhythm and cardiovascular risk factors. Plat et al, in 1999, observed 9 healthy volunteers who underwent cortisol suppression through metyrapone and were consequently treated with hydrocortisone replacement therapy. They reported that a delayed cortisol rhythm, characterized by a relative cortisol excess in the evening, was associated with an increase in glucose and insulin concentrations, testifying to a worsening in glucose metabolism (102). Ceccato et al analyzed the circadian rhythm of 120 adult volunteers during an ordinary weekday, collecting 7 saliva samples during the day. They observed that late-night salivary cortisol (LNSC) was higher among hypertensive patients, while LNSC and the ratio between LNSC and morning salivary cortisol were increased among patients with metabolic syndrome. Moreover, an age-related increase only for evening cortisol and LNSC was reported, while the total daily cortisol secretion was not different in younger patients (103). This could be considered, consequently, as a marker of impaired circadian rhythm, which shows a blunt decline in elderly people. The same group later observed that LNSC in people with diabetes or obesity was higher than that of a healthy control group (104). Therefore, even patients without overt endocrine diseases may present some circadian cortisol impairment which can contribute to increased cardiovascular risks or metabolic comorbidities. Likewise, in pathological contexts secondary to cortisol excess conditions (ie, Cushing syndrome), the total disruption of GC circadian rhythm is one of the main hallmarks and is key in enhancing the well-known metabolic and cardiovascular complications of this disease (105). Recently, a significant disruption in several clock genes’ expression in innate and adaptive immune cell subpopulations has been demonstrated in patients with Cushing syndrome, suggesting new potential mechanisms that could influence the increased risk of infections in this syndrome (106).

Much less is known regarding mineralocorticoids’ diurnal rhythm: the aldosterone secretion seems to have a morning peak around 8 Am, with a decrease at nighttime (107-111). It is, therefore, likely that mineralocorticoids fluctuations might follow a diurnal rhythm that is finely intertwined with other factors, such as sleep schedule variation (112).

### Glucocorticoid and Mineralocorticoid Hormones Therapy

Patients with adrenal insufficiency require lifelong GC replacement therapy mimicking the natural circadian pattern of cortisol production, and avoiding under- or over-replacement (113).

According to the Endocrine Society guidelines for primary adrenal insufficiency (PAI), 15 to 25 mg of hydrocortisone or 20 to 35 mg of cortisone acetate should be administered. The total daily dose should be divided into 2 to 3 doses per day, giving the largest amount in the morning, then one at lunch and one in the late afternoon (62). Even then, the replication of the physiological cortisol circadian rhythm is only partial. In fact, serum cortisol reaches a peak after hydrocortisone assumption, dropping below normal before the next dose (114). An alternative drug is cortisone acetate, using which the serum cortisol peak after the oral assumption seems to be lower; moreover, serum levels tend to have a slower decline than on hydrocortisone therapy (114). However, none of these regimens accurately replicate the cortisol circadian rhythm (115). Relatively novel dual-release hydrocortisone is made of an immediate releasing coat, plus an extended-release core. The final effect is a morning peak of GC, followed by a gradual decrease during the rest of the day. Total daily cortisol exposure is estimated to be 20% lower compared to that obtained with conventional hydrocortisone therapy (116) and this seems associated with an improvement in the quality of life and metabolic parameters of patients with adrenal insufficiency (117) (Fig. 1B).

In contrast to the aforementioned arguments, in cases of congenital adrenal hyperplasia (CAH) the administration of GC differs, as the aim of the treatment is to replace the cortisol deficiency, while restoring the negative feedback on pituitary adrenocorticotropic hormone (ACTH) to avoid androgen excess at the same time (118, 119). High-potency GC like prednisolone or dexamethasone are effective in suppressing ACTH excess and their long t_1/2_ allows a twice or once-daily administration, provided that the risk of long-term consequences of the excess of treatment, like iatrogenic Cushing syndrome, is carefully considered (120, 121). Therefore, immediate-release hydrocortisone is usually the preferred option in childhood and adulthood (118), but it might be insufficient to provide adequate adrenal androgen suppression overnight, requiring supraphysiological GC doses (122). In 2021, a new formulation of hydrocortisone (Efmody) has been approved, consisting of tablets with a coating layer that provides a delayed absorption of the drug. A twice-daily administration is suggested, giving two-thirds of the daily dose at bedtime, while the remaining one-third is given in the morning, ensuring cortisol supply during the day. The administration of the higher dose of GC at bedtime might appear to contrast with the physiological circadian cortisol secretion; however, the particular PK of this hydrocortisone formulation allows a reduction of the morning ACTH surge. This PK is indeed able to replicate the natural diurnal cortisol cycle, allowing a reduction of GC dose as well (123, 124) (Table 2). Additionally, the novel oral corticotropin-releasing factor type 1 receptor antagonist Crinecerfont has been found to effectively control adrenal-derived androgen excess allowing for reduction of glucocorticoid replacement and improvement in physiology-mimicking administration timing (125).

**Table 2.** Glucocorticoid formulations

GC formulation	Description	Indication	Administration schedule	Pros	Cons
**Hydrocortisone**	Short-acting GC with peak serum levels achieved 30-60 minutes post-oral administration	Adrenal Insufficiency (GC replacement mimicking cortisol circadian pattern), CAH (GC replacement, adequate ACTH suppression to avoid androgen excess)	2-3 times/day	Approximates physiology, rapid onset	Requires multiple daily doses to maintain levels; incomplete replication of circadian rhythm; risk of over- or under-replacement between doses
**Cortisone acetate**	Pro-drug converted to cortisol in the liver	Adrenal Insufficiency (GC replacement mimicking cortisol circadian pattern)	2-3 times/day	Slower decline in cortisol levels compared to hydrocortisone, easily available, cheap	Slower activation through liver metabolism; requires multiple doses to approximate circadian cortisol levels
**Dual-release hydrocortisone (Plenadren)**	Combines immediate- and extended-release components	Adrenal Insufficiency (GC replacement mimicking cortisol circadian pattern)	Once daily	Better mimics physiological cortisol rhythm, reduces metabolic side effects, improves the quality of life	Costly, may not provide adequate late-night cortisol levels in some patients
**Delayed-release hydrocortisone (Efmody)**	Coated tablet with delayed absorption for bedtime dosing and additional morning dose	CAH (GC replacement, restore the negative feedback on ACTH to avoid androgen excess)	Twice daily	Closely mimics physiology, sustained ACTH suppression, allows GC dose reduction	Costly
**Prednisolone/Prednisone**	Intermediate-acting GC with longer half-life (12-36 hours)	CAH (GC replacement, restore the negative feedback on ACTH to avoid androgen excess)	1-2 times/day	Sustained ACTH suppression, easily available, cheap	Risk of overtreatment and iatrogenic Cushing's syndrome, does not align with natural circadian cortisol patterns
**Dexamethasone**	Long-acting GC with a half-life of 36-72 hours	CAH (GC replacement, restore the negative feedback on ACTH to avoid androgen excess)	Once daily	Long half-life allows for once-daily dosing, sustained ACTH suppression, easily available, cheap	Risk of overtreatment and iatrogenic Cushing's syndrome, does not align with natural circadian cortisol patterns

Abbreviations: ACTH, adrenocorticotropic hormone; CAH, congenital adrenal hyperplasia; GC, glucocorticoid.

The synthetic mineralocorticoid fludrocortisone (9α-fludrocortisol) is used as additional replacement therapy in primary adrenal insufficiency (PAI), on top of hydrocortisone as needed (62). Its plasmatic peak is 2 hours after oral administration, and is usually taken once daily, in the morning, due to the morning peak of aldosterone.

In conclusion, the timing of GC administration is essential to avoid the disruption of cortisol circadian rhythm with potential adverse metabolic and cardiovascular complications, with novel formulations better mimicking physiology. Regarding mineralocorticoid administration, as well-established by the current guidelines regarding adrenal insufficiency and mineralocorticoid replacement therapy (62), the best timing of administration of fludrocortisone is once daily, around 8 Am, in order to respect the circadian rhythm of aldosterone reported by most (but not all) studies.

## Testosterone

### Testosterone Diurnal Rhythm

Testosterone (T) exhibits a diurnal pattern (126) (Fig. 1A), presenting a peak around 8 Am and a progressive decline with nadir in the late afternoon and early evening. Nocturnal rises in T are related to REM sleep (127) and responsible for sleep-related erections (128). This pattern, blunted in hypogonadal males (129), depends on gonadotropin-releasing hormone (GnRH) and luteinizing hormone (LH) SCN-driven pulsatile release (130), and on cyclical variation of the testicular sensitivity to LH (131). In particular, different LH pulse amplitudes and frequencies regulate testosterone rhythm. Although such rhythm cannot be attributed to an endogenous circadian regulation, as it mostly associates with the sleep/wake cycle and not the time of the day (132), LH pulses somewhat show a circadian variation, with lower pulse frequency and higher pulse amplitude at night (133). With aging, this rhythm becomes less apparent, due to smaller and more frequent LH pulses secreted at night with respect to younger males, altered T feedback on gonadotropin production, as well as decreased testicular responsiveness and defect in Leydig cell steroidogenesis (134-136). Night-shift workers with sleep disorders have increased hypogonadal symptoms (137), with a significant impairment of erectile function (138) and semen parameters (139). Given the role of T in glucose homeostasis and lipid metabolism, disruption in diurnal rhythm and reduced level of T may contribute to adverse metabolic outcomes, such as reduced insulin sensitivity, metabolic syndrome, and body composition changes, particularly a reduction in lean body mass and an increase in visceral fat mass (140-142). This last shift enhances the aromatase activity in adipocytes, converting T into estradiol, which further exacerbates T deficiency by suppressing GnRH secretion, perpetuating a self-reinforcing cycle and amplifying metabolic dysfunction in hypogonadal males (143).

Day-to-day and 3-month measurements of total T, free T, and bioavailable T demonstrated that significant intra-individual ultradian variation exists, brought on by pulsatile release, especially for LH, making it difficult to rely on one measurement only (144). Moreover, compared to nonfasting levels, fasting men have significantly higher serum T levels (145), raising an important issue with regard to the optimal conditions under which T should be assessed in men ie, in fasting state, around 8 to 10 Am, in 2 separate blood samples, as recommended by the most recent guidelines on hypogonadism (63, 64) (Table 1).

### Testosterone Therapy

Over the years, testosterone replacement therapy (TRT) has evolved, leading to the development of numerous preparations and formulations. These advancements, which offer a variable time of onset of effects, have been aimed at enhancing PK and patient compliance (146, 147). Oral, transdermal, and IM modes of administration exist, although only the last two are the most commonly employed, as oral administration is burdened by a significant first-pass effect, thereby reducing its bioavailability (148).

Transdermal application of T is a noninvasive, easy-to-apply formulation, which guarantees a quick reversal after discontinuation and levels of T that can follow the physiological diurnal rhythm. Most of the approved topical TRT products are administered daily and show a T_max_ at 2 to 4 hours after application, then gradually declining, mirroring the normal T diurnal variation (149). T patches were among the first formulations used for TRT, and in recent years, they have been replaced by easier, more patient-friendly gels or solutions. When T is applied to the skin as a hydroalcoholic gel, it dries rapidly, and the steroid is absorbed into the stratum corneum, which serves as a reservoir, releasing T into the circulation slowly over several hours, thereby preventing supraphysiological peaks (150).

Intramuscular (IM) preparations of T are available, thanks to the esterification of the 17β carbon of natural T, which otherwise would have lower solubility and an approximate t_1/2_ of 10 minutes when injected. Approved IM preparations are T cypionate, T enanthate, T propionate, and T undecanoate. They are injected at a frequency of once weekly to once every 14 weeks, depending on the formulation, with the most rapid and significant peaks in serum T levels reached within days of the injection when administering short-acting formulations (151). The PK of short-acting and very short-acting formulations, such as propionate, cypionate, and enanthate parental T formulations, might cause significant fluctuations in serum T concentrations over the period of administration (151, 152). Supraphysiological peaks might increase the risk of adverse effects, especially polycythemia, but even changes in libido and mood, particularly in fragile populations (152, 153). These effects are the same experienced by anabolic steroid androgen abusers and include the inhibition of the gonadotropic axis responsible for hypogonadotropic hypogonadism and infertility in men and masculinization, hirsutism, and anovulation in women. On the other hand, long-acting parental T formulation, such as T undecanoate, seems to restore serum T concentrations in the normal range, with a better safety profile and without showing supratherapeutic peaks (154), and with fewer administrations needed, a fact that might relieve the patient from having daily reminders of having a chronic disease (155). Using longer-lasting formulations and increasing the interval between administrations is a common strategy, particularly when hematocrit and T levels are at the upper limit of the reference range or when LH is suppressed, to overcome supraphysiological levels caused by IM injections. Moreover, it has been suggested that T undecanoate, due to its PK, when employed in the gender-affirming hormonal therapy of assigned females at birth transgender patients, may provide a more sustained gonadotropin suppression and result in less aromatization to estradiol, compared with gel formulations (156) (Table 3).

**Table 3.** Testosterone formulations

Testosterone formulation	Description	Indication	Administration schedule	Pros	Cons
**Transdermal gel**	Noninvasive formulation absorbed through the skin, mimics physiological diurnal testosterone rhythm	Hypogonadism, gender-affirming hormone therapy	Once daily	Easy to use, mimics diurnal rhythm, rapid reversal after discontinuation, avoids supraphysiological peaks	Requires daily application, potential skin irritation, risk of transference to others
**Testosterone propionate**	Very short-acting injectable testosterone ester with rapid absorption and elimination	Hypogonadism, gender-affirming hormone therapy	Every 3-4 days	Rapid onset of action, effective for short-term use, cheap	Significant fluctuations in serum testosterone, risk of supraphysiological levels, requires frequent injections, no diurnal rhythm mimicking
**Testosterone Cypionate/Enanthate**	Injectable, intermediate-acting testosterone esters with peak levels in 2-3 days post-injection	Hypogonadism, gender-affirming hormone therapy	Every 1-2 weeks	Effective for sustained testosterone levels, fewer administrations needed compared to gels, cheap	Significant fluctuations in serum testosterone, risk of supraphysiological levels, requires injections, no diurnal rhythm mimicking
**Testosterone Undecanoate**	Long-acting injectable testosterone ester with extended release over several weeks	Hypogonadism, gender-affirming hormone therapy	Every 10-14 weeks	Minimal peaks and troughs, fewer administrations, better safety profile than short-acting esters	No diurnal rhythm mimicking, costly

In regards to chronotherapy, morning administration of transdermal formulations is theoretically more advisable, as this better mimics the physiological diurnal rhythm of testosterone. However, since bedtime administration may be associated with higher patients’ compliance, more studies are needed to clarify if this may represent a feasible and effective alternative. Long-acting, depot preparations offer the significant benefit of bimonthly or quarterly administration, but they inherently lack the ability to accommodate diurnal rhythms, and there is no guidance on the daily timing of their administration. While this characteristic might make them less suitable for chronotherapy, the practical benefits of less frequent dosing often outweigh this limitation (157) (Fig. 1B).

## Estrogen and Progesterone

### Estrogen and Progesterone Diurnal Rhythm

Most female reproductive hormones follow a circadian rhythmicity (158), and mutations in clock genes lead, in turn, to alterations in fertility in female mice (159, 160). It has been suggested that disruptions in the mechanisms regulating these circadian fluctuations could also play a role in disturbances of the hypothalamic-pituitary-gonadal axis in women (161). For instance, in human granulosa cells, the diurnal rhythm of clock gene expression attenuates with age, suggesting that the reduced ovarian reserve in older women may be partially attributed to ovarian circadian dysrhythmia resulting from the aging process (162).

The link between circadian rhythms and estrogen has been clearly demonstrated (163). Estrogen receptors (ERs) are expressed in the SCN (164), and, in turn, clock genes are able to influence the activity of the ER-α by regulating its transcriptional activity (165). The observed diurnal rhythm of free estradiol has a complex temporal organization, consisting of 2 main components: a diurnal cycle with an asymmetric peak and ultradian harmonics in the interval between 6 and 12 hours. On the other hand, progesterone release occurs in a periodic (diurnal and ultradian) fashion as well as in a pulsatile mode (132, 166). The attainment of maximal serum progesterone concentrations seems to occur approximately at 6 Pm (166).

It has been shown that the regulation of the female reproductive hormones by the circadian clock also depends on the menstrual phase (158). A 24-hour circadian rhythm during the follicular phase seems to occur for estradiol and progesterone; however, this circadian pattern does not seem to be maintained during the luteal phase, although data are controversial (167). Salivary estradiol tends to peak early in the morning, and such peak tends to occur later in the morning in the menstrual phase of the ovarian cycle than in the late follicular/peri-ovulatory phase (168). The loss of rhythmicity in progesterone during the luteal phase is likely due to a shift in the primary source of progesterone from the adrenal cortex during the follicular phase to the corpus luteum during the luteal phase, whose secretion may not be rhythmic.

The disruption of the circadian rhythm of the ovary appears to be associated with the development of hyperandrogenism, polycystic ovary syndrome (PCOS) and alterations of the hypothalamus-pituitary-gonadal axis. Specifically, evidence shows that in female night-shift workers, PCOS is more common, and there is evidence of genome-wide chronodisruption in granulosa cells (169). Indeed, it appears that women with PCOS have reduced peripheral expression of CLOCK and BMAL1, which alters steroidogenic genes causing hyperandrogenism (170). Interestingly, infertile women with PCOS more commonly report sleep disturbances than those with unexplained infertility (171). However, given the close relationship that PCOS has with obesity and insulin resistance (172) and that these 2 entities are, in turn, characterized by alterations in circadian rhythm and sleep disturbances (173), it remains to be clarified whether these alterations are found independently in PCOS or they are the result of underlying obesity and insulin resistance.

### Estrogen/Progesterone and Combined Estroprogestinic Therapy

Estrogens can be prescribed for several therapeutic purposes, such as hormone replacement therapy (HRT) for postmenopausal or hypogonadotropic women, contraception (174), menstrual abnormalities (175), and gender incongruence in assigned male at birth (AMAB) patients (176). Different routes of administration exist for estrogens (oral, transdermal, or vaginal), and they have different effects on clinical outcomes (177). Transdermal administration of 17-β-estradiol is the route that best mimics physiological serum concentrations of estradiol and offers a better safety profile compared to oral formulations (178). In fact, transdermal administration avoids the hepatic first-pass effect, minimizing the impact of estrogen on coagulation, and has little impact on the lipid profile, markers of inflammation and blood pressure (179). Transdermal 17-β-estradiol has also been shown more effective in achieving peak bone mineral density and reducing markers of bone resorption than oral ethinylestradiol (EE) (180). On the other hand, several progestins are available and should be prescribed alone in some cases or as a component of combined HRT (181). Progestins and estrogens are metabolized by hepatic cytochromes, in particular CYP3A, which contribute significantly to their high hepatic first-pass effect, reducing their circulating levels after oral administration compared to vaginal and transdermal use. This also explains the high drug-drug interaction that estro-progestins undergo (182).

In HRT, estrogen therapy should be accompanied by an adequate dose of progestin (administered continuously or sequentially) to prevent endometrial hyperplasia and cancer in non-hysterectomized women. Hysterectomized women, on the other hand, should be prescribed estrogen-only HRT (183). AMAB patients also are not prescribed progestin, but estrogen is combined with an antiandrogen (176).

The goal of combined hormonal contraceptives is not hormone replacement, but contraception achieved by suppression of the gonadotropic axis. Therefore, dosages of estrogen and progestin are higher than in HRT therapeutic schemes (184). Most combined hormonal contraceptive formulations contain EE; however, due to their potentially safer profile, natural estrogens such as 17-β-estradiol or its ester valerate are increasingly prescribed (185). Regardless of the agent prescribed, estrogen and progestin therapy must be taken at the right times to maintain appropriate circulating levels. Available formulations include oral (estrogen, progestin, or both) to be taken at the same time each day; vaginal (EE +etonogestrel) to be inserted and left in the vagina for 3 consecutive weeks; and transdermal (estradiol hemihydrate or ethinylestradiol + norelgestromin) to be changed weekly. Ideally, administration should mimic normal ovarian function: transdermal and vaginal routes delivering estradiol 0.100 mg daily are the first rudimentary step in this direction. These formulations mimic the daily ovarian production rate of estradiol and achieve average serum estradiol levels of 100 pg/mL (178). Regarding daily formulations, such as pills, current data does not sufficiently support the recommendation of taking the therapy at a specific time of day (186). Given the extreme complexity and variability of both estrogen and progesterone circadian and ultradian rhythms, it is unlikely that any specific administration timing may prove better than others (Fig. 1B).

## Antidiuretic Hormone

### Antidiuretic Hormone Diurnal Rhythm and Therapy

Healthy individuals have a nocturnal decrease in urine output due to increased plasma antidiuretic hormone (ADH) levels at night (187). In fact, children with enuresis and nocturnal polyuria may lack the physiologic increase in ADH levels during sleep, and treatment with the ADH synthetic analogue desmopressin might be necessary to restore this rhythm (188). Furthermore, there is evidence for a diurnal rhythm in copeptin—the C-terminal part of the ADH precursor—release, similar to the rhythm of ADH, with higher levels at night and early morning and lower levels in the late afternoon (189) (Fig. 1A).

Desmopressin acetate, also known as 1-desamino-8-D-AVP (DDAVP), is the synthetic analogue of ADH. The antidiuretic properties of desmopressin have led to its use in polyuric conditions including, among others, arginine vasopressin deficiency (AVP-D, formerly known as central diabetes insipidus) (190, 191). Desmopressin can be administered intranasally, orally, subcutaneously, or intravenously. The absorption of desmopressin acetate through the nasal mucosa is highly variable, secondary to mucosal inflammation or congestion, and the duration of the antidiuretic effect is more variable than that of the oral administration, ranging from 5 to 21 hours (192). The oral form is generally preferred to the nasal spray due to the ease of administration, the more stable PK, and the preserved efficacy even in cases of nasal congestion or infection. The peak is reached within 2 hours of ingestion, and the antidiuretic action lasts for 6 to 12 hours (193). Patients with complete AVP-D usually need a dose 2 to 3 times daily, whereas in patients with partial AVP-D, a single bedtime dose might be sufficient to control nicturia (194).

Desmopressin is dosed empirically, and ADH is not routinely measured. According to previous evidence, the first dose of desmopressin is usually given at bedtime to initially reduce nicturia and to respect the circadian rhythm of ADH. If polyuria and polydipsia persist during the day, a daytime dose might be necessary (194) (Fig. 1B).

## Growth Hormone

### Growth Hormone Diurnal Rhythm and Therapy

The SCN acts on somatostatinergic neurons and growth hormone (GH)-releasing hormones (GHRH) to synchronize the GH rhythm and the GH/ insulin-like growth factor 1 (IGF-1) axis with the light-dark cycle (195). In particular, the diurnal pattern of GH, which develops after puberty, demonstrates a major peak at late night/early morning, and several peaks during the light hours of the day, but with quite large individual difference (196) (Fig. 1A). GH levels are increased during sleep, with a major increase occurring soon after sleep onset, irrespective of the time of day that sleep takes place (197). Moreover, with aging, there is a decrease in the pulsatile release of GH (198). Despite the strict correlation between GH secretion and the sleep/awake cycle, whether the GH synthesis is really subjected to circadian regulation is not completely understood. Vakili et al (199) observed on transgenic mice, under strict light-dark cycles, that human GH RNA expression oscillates over a 24-hour period, suggesting a circadian pattern that was not confirmed when analyzing the mouse GH expression. Interestingly, they provide evidence that Bmal1 and CLOCK might transactivate the human GH promoter as well, confirming the likely influence of the circadian machinery on GH secretion.

The influence of Bmal1 in the GH control was recently explored in Bmal1 knock-out mice, showing disruption of GH pulsatility and of IGF-1 hepatic secretion (200) reinforcing the evidence of a strict connection between GH secretion and clock genes. However, further studies in human subjects are required for a deep understanding of the circadian control of the GH/IGF-1 axis.

Although animal models have shown that pulsatile administration of GH resulted in better growth and greater IGF-1 production, human studies have failed to show clinically meaningful changes compared with daily recombinant human GH (rhGH) injections.

GH deficiency (GHD) results from various hereditary or acquired causes, which might be isolated or combined with other pituitary hormone deficiencies (201). Nowadays, rhGH therapy is administered as daily subcutaneous injections and has been shown beneficial in the treatment of GHD in children (CGHD) and adults (AGHD) (202). Notwithstanding, the debate on PK and PD of different GH preparations continues and, currently, multiple preparations of long-acting rhGH are available or under study, allowing for decreased rhGH injection frequency from daily to weekly or monthly (203). Bedtime administration is usually recommended and was proven better than morning administration in terms of ensuring more physiological GH profiles (204). However, current guidelines do not specify a best timing (202, 205) (Fig. 1B). When considering the measurement of IGF-1, as a surrogate of GH/IGF-1 axis, in light of the abovementioned evidence regarding its diurnal rhythm and potential alterations on IGF-1 concentrations by food intake (206, 207), a morning plasma sample of IGF-1, with fasting, may be suggested (Table 1).

## Insulin

### Insulin Circadian Rhythm

Insulin secretion is regulated by the intricate interplay between nutrient sensing, gastro-entero-pancreatic hormones, and the autonomic nervous system. Glucose, along with certain other sugars metabolized by the pancreatic islets, plays a pivotal role in stimulating insulin release. Consequently, both basal and peak insulin levels are closely correlated with glucose concentrations and the timing of meals (208). During prolonged fasting, glucose and insulin levels decrease further; however, they typically remain measurable within the range of 2 to 5 mU/mL (209). Insulin secretion exhibits a robust circadian rhythmicity, with serum insulin concentration increasing from a nadir between midnight and 6 Am and peaking between noon and 6 Pm, which is critical for maintaining normal glucose homeostasis (Fig. 1A). This has been demonstrated by one study that, through the application of a circadian misalignment protocol, clearly showed that the diurnal rhythm of β-cell sensitivity is intrinsically regulated by the circadian clock (210). By reversing participants' behavioral cycles, the authors successfully isolated the effects of the circadian phase from external factors such as the sleep/wake and feeding/fasting cycles. The findings revealed a significant reduction in glucose tolerance during the biological evening compared to the biological morning, even under conditions of behavioral misalignment. This reduction, attributed to a 27% decrease in early-phase insulin secretion, provides strong evidence that the diurnal variation in β-cell sensitivity is driven by the circadian clock itself, independent of external behavioral influences. This circadian rhythm may contribute to the improved glucose tolerance observed in the morning hours relative to the evening.

Insulin secretion also exhibits ultradian rhythms (period of ∼40 minutes) and high-frequency rhythms (period of ∼4 minutes) (211), being intricately regulated by the pancreatic circadian clock, which operates autonomously in the pancreas and aligns with central signals from the brain's suprachiasmatic nucleus. This clock controls the transcription of genes critical for insulin biosynthesis, transport, and glucose-stimulated insulin release, driven by key proteins such as CLOCK and BMAL1 (173). Studies have demonstrated circadian rhythms in insulin secretion in rodent pancreatic islets (212), and similar mechanisms have been confirmed in human islets (213). Disruption of this clock impairs insulin secretion, highlighting its essential role in maintaining glucose homeostasis and emphasizing the broader metabolic implications of circadian misalignment (214). Furthermore, disruption of the circadian regulation of insulin secretion is associated with reduced peripheral glucose uptake and an increased risk of T2DM. Findings from a study in 2020 in rodent studies and in vitro experiments have provided evidence that the deletion of circadian clock genes, such as *cry1* and *cry2*, leads to a disruption in the circadian rhythmicity of both insulin and glucagon secretion (215).

Interestingly, insulin itself directly regulates the circadian clock in adipose tissue: researchers studying participants with obesity and no T2DM found that postprandial insulin levels significantly altered the expression of key clock genes like *PER2*, suggesting that insulin-dependent mechanisms play a role in regulating the internal clock of adipose tissue (216). These findings highlight the multifaceted role of insulin, not only in affecting metabolic processes but also in serving as a *zeitgeber* for local clocks in adipose tissue. This regulation is particularly relevant as it indicates that meal time can influence circadian peripheral tissue rhythms (217), with the wrong timing of food intake potentially leading to metabolic disorders such as obesity and T2DM (218).

### Insulin Therapy

Since the introduction of exogenous insulins, insulin therapy has become a cornerstone in the management of diabetes, especially insulin-dependent forms, where different formulations can be used to meet patients' individual needs. These formulations include rapid-acting, short-acting, intermediate-acting, and long-acting insulins, each designed to mimic the body's natural insulin response to meals (219).

The timing of long-acting insulin injections depends on factors like blood glucose levels, food consumption, exercise, and the types of insulin adopted. The optimal timing for administering long-acting insulin in clinical practice remains an area of ongoing investigation. While an evening administration is commonly employed, a 2015 study by Porcellati et al directly compared the PK and PD of insulin glargine after evening vs morning subcutaneous injection in individuals with T2DM (220), showing that the pharmacodynamic profile of insulin glargine differed based on the timing of administration. With morning dosing, insulin activity was greater during the initial 0 to 12-hour period. Conversely, evening administration resulted in enhanced activity in the subsequent 12- to 24-hour window post-injection. However, glargine PK, plasma C-peptide levels, and overall 24-hour PD were comparable between the 2 administration time points.

These results highlight that the differences in insulin activity are driven by circadian variations in insulin sensitivity, which is typically lower during the night and early morning hours compared to the afternoon, rather than an inherent property of insulin glargine itself. This study also highlights the need to consider the role of diurnal rhythms in insulin action when optimizing basal insulin therapy in patients with T2DM. For instance, morning administration of long-acting insulin is recommended for patients who receive glucocorticoids in the morning and experience glycemic spikes in the afternoon (221). In summary, evening administration of long-acting insulin is recommended for patients with T2DM not taking glucocorticoids, although it is not mandatory. For optimal blood glucose management, it is important to take long-acting insulin at the same time(s) each day, whether in the morning or at another consistent time (Fig. 1B).

Recently, there has been a growing interest in weekly insulin formulations, which are currently under investigation (222). These long-acting options aim to simplify diabetes management by reducing the frequency of injections while maintaining effective glycemic control. The development of these innovative formulations reflects ongoing advancements in insulin therapy, emphasizing the importance of individualized treatment plans tailored to each patient's lifestyle and metabolic needs. While there are theoretical concerns about the potential negative effects of not mimicking circadian rhythms with these long-acting formulations, it is reasonable to conclude that the benefits of increased convenience and improved adherence outweigh these potential drawbacks.

## Leptin

### Leptin Diurnal Rhythm and Therapy

Leptin is an anorectic hormone primarily produced by adipocytes, serving as a key indicator of the body's energy status. Beyond fluctuations dependent on food intake, leptin plasma levels exhibit a distinctive 24-hour rhythm, influenced by both circadian and sleep-related factors. In particular, Brandenberger and colleagues demonstrated that in healthy men under constant conditions (continuous enteral nutrition and bed rest in controlled chambers), leptin levels peaked at the start of the resting phase, with a maximum during nocturnal sleep in the early morning (109.9% of the 24-hour mean) and a nadir in the late afternoon. When sleep was shifted to the daytime, leptin levels peaked during diurnal sleep but with reduced variation (223). This rhythm, characteristic of humans as diurnal animals, aligns with the early dark phase and correlates closely with body temperature rhythms, glucose levels, and insulin fluctuations, emphasizing leptin's role in circadian metabolic and thermoregulatory regulation (Fig. 1A). Leptin crosses the blood-brain barrier (BBB) by a saturable transport mechanism, and this transport has a circadian rhythm both at the BBB and the blood-spinal cord barrier. Centrally, it functions by inhibiting neuropeptide Y (NPY) and agouti-related peptide (AgRP) neurons while stimulating pro-opiomelanocortin (POMC) and Cocaine- and Amphetamine-Regulated Transcript (CART) neurons. The removal of the SCN in rodents disrupts the rhythmicity of leptin secretion (224), further confirming that leptin expression is influenced not only by food intake but also by circadian oscillators (225).

Interestingly, adipose tissue is regulated by an endogenous circadian clock, and feeding contributes to regulating this clock with external time cues (226). Although the specific mechanisms of such regulation are still not fully understood, leptin has been suggested to play a significant role. The role of leptin in modulating peripheral clocks has been demonstrated through studies conducted on leptin-deficient mice (ob/ob), which exhibited altered expression profiles of clock genes in the liver and white adipose tissue (227). Additionally, disrupted leptin signaling in ob/ob or db/db mice (which have mutated receptors) led to disturbances in the rhythmic oscillations of plasma glucose levels. Spectral analysis revealed 2 components (periodicities: 24 and 12 hours) with higher relative amplitudes in lean than in subjects with obesity suggesting that attenuated or altered circadian rhythmicity might play a role in the development of leptin resistance and obesity (228).

Metreleptin is a recombinant form of the hormone leptin, which is primarily indicated for individuals with congenital leptin deficiency and certain forms of lipodystrophy. The administration of leptin is carried out subcutaneously, with dosing tailored to the individual's body weight and specific condition. Leptin is typically administered every 24 hours to maintain stable serum levels. There is no strict requirement regarding the specific time of day it must be administered (eg, morning or evening), but consistency is key. Administering it at the same time each day helps maintain stable leptin levels and improves the effectiveness of the treatment. (Figure 1B).

## Glucagon-Like Peptide 1

### Glucagon-Like Peptide 1 Diurnal Rhythm

Glucagon-like peptide 1 (GLP-1) is produced both peripherally in the L cells of the small intestine and the α cells of the pancreas, as well as centrally in several brain regions including the nucleus of the solitary tract, the dorsal and central reticular nuclei, the paraventricular nucleus (PVN), the dorsomedial hypothalamic nucleus (DMH), and the arcuate nucleus (ARC) (229). GLP-1 plays a role in reducing glucagon release and promoting insulin secretion in response to elevated postprandial blood glucose levels, contributing to the sensation of fullness by slowing gastric emptying and influencing central appetite regulation centers (230).

Evidence for the diurnal rhythm of GLP-1 has been reported by Zilstorff et al. The authors conducted a study involving 24 healthy men with regular sleep schedules to examine the rhythms of incretins (231). The participants were monitored in a hospital ward with standardized food intake, and blood samples were collected every 3 hours. Both GLP-1 and gastric inhibitory polypeptide (GIP) exhibited a distinct diurnal rhythm, peaking during the day, with GLP-1 and GIP peaking between 5:30 and 6 Pm, and reaching their lowest levels at night (Fig. 1A).

In vitro studies conducted on human and rat L cell lines have suggested that GLP-1 exhibits a circadian secretion pattern: both cell lines demonstrated, in fact, an autonomous cellular rhythm in the expression of Bmal1, with GLP-1 secretion following the same pattern (232). Suppression of Bmal1 was associated with a reduction in GLP-1 release, and primary intestinal cultures from Bmal1 knock-out mice showed decreased GLP-1 secretion (233).

### Glucagon-Like Peptide 1 Receptor Agonist Therapy

The timing of GLP-1 receptor agonists (GLP-1RA) administration can significantly impact their effectiveness in managing obesity and diabetes. While the optimal timing may vary depending on the specific GLP-1RA and individual patient factors, there are general guidelines based on current research and clinical practice.

For daily administered GLP-1RAs, such as liraglutide, it is often recommended to take them at the same time each day to maintain consistent blood levels. Some studies suggest that taking these medications before breakfast can help with appetite suppression throughout the day (234). Although administering the medication later in the day to mimic the late afternoon peak would indeed be more in time with the described circadianity, the reduced hunger suppression being experienced right before the subsequent administration the morning after makes this timing impractical.

Oral semaglutide, indicated for diabetes, should be taken on an empty stomach upon waking up in the morning. After taking semaglutide, it is crucial to wait at least 30 minutes before eating, drinking, or taking any other oral medications (235). In 2023, a randomized trial investigated the effect of various dosing schedules on the PK of oral semaglutide in healthy subjects. Compared to an overnight pre-dose fast followed by a 30-minute post-dose fast (reference arm), shorter pre-dose fasting times of 2, 4, or 6 hours followed by a 30-minute post-dose fast resulted in significantly lower semaglutide exposure, as measured by the area under the curve (AUC) and peak concentration (C_max_) after the tenth dose (236). These findings indicate that while shorter pre-dose fasting times reduce semaglutide absorption, maintaining an overnight fast before dosing and at least a 30-minute fast after dosing allows for adequate drug exposure, consistent with current prescribing recommendations.

For weekly GLP-1RAs like semaglutide and dulaglutide, or on the contrary, for those with a short t_1/2_ such as exenatide, the administration cannot intrinsically respect circadianity. For example, exenatide should be taken within 60 minutes before the 2 main meals of the day, at least 6 hours apart. This timing helps maximize its effect on postprandial blood glucose levels (236).

Ultimately, the best time to administer GLP-1RAs may vary based on individual lifestyle, meal patterns, and side effects. Patients should work closely with their healthcare provider to determine the most suitable timing for their specific situation and to ensure optimal management of obesity and diabetes (Fig. 1B).

## Conclusions

Recent technological advancements are significantly influencing the drug development process, with profound implications for the pharmacotherapy of various disorders. A central goal of HRT and novel pharmaceuticals is to prevent morbidity and mortality and to improve quality of life. Indeed, optimizing drug administration timing to coincide with the body's natural hormonal circadian rhythms and individual chronotype is increasingly recognized as an effective strategy.

However, the advent of long-lasting or depot formulations has shifted the priority from chronotherapy in some cases. These formulations enhance patient compliance and can sometimes increase medication efficacy. Therefore, it is essential to find the right balance between the benefits of chronotherapy and the advantages of user-friendly, long-acting medications. This balance should be tailored to the specific disease being treated and the importance of circadian rhythms for the particular hormone or treatment.

Providing explicit guidance on the timing of medication intake, such as utilizing charts, offers a strategic approach to fostering rational, compliance-oriented chronotherapy, thereby aiding in determining the most appropriate drug dosing schedule in each context.

## Abbreviations

ACTH: adrenocorticotropic hormone
ADH: antidiuretic hormone
AMAB: assigned male at birth
AVP-D: arginine vasopressin deficiency
CAH: congenital adrenal hyperplasia
EE: ethinylestradiol
fT4: free thyroxine
GC: glucocorticoids
GH: growth hormone
GLP-1: glucagon-like peptide 1
GLP-1RA: glucagon-like peptide-1 receptor agonist
GnRH: gonadotropin-releasing hormone
HRT: hormone replacement therapy
IGF-1: insulin-like growth factor 1
IM: intramuscular
LH: luteinizing hormone
LNSC: late-night salivary cortisol
L-T4: levothyroxine
PCOS: polycystic ovary syndrome
PD: pharmacodynamics
PK: pharmacokinetics
PTH: parathyroid hormone
SCN: suprachiasmatic nuclei
T: testosterone
T_1/2_: half-life
T2DM: type 2 diabetes
T3: triiodothyronine
T4: thyroxine
T_max_: peak exposure time
TRT: testosterone replacement therapy
TSH: thyrotropin (thyroid-stimulating hormone)
